# Rapid translation of clinical guidelines into executable knowledge: A case study of COVID‐19 and online demonstration

**DOI:** 10.1002/lrh2.10236

**Published:** 2020-07-14

**Authors:** John Fox, Omar Khan, Hywel Curtis, Andrew Wright, Carla Pal, Neil Cockburn, Jennifer Cooper, Joht S. Chandan, Krishnarajah Nirantharakumar

**Affiliations:** ^1^ OpenClinical London UK; ^2^ Institute of Digital Healthcare, Faculty of Science Engineering and Medicine University of Warwick Coventry UK; ^3^ Dynamic Technologies Ltd. Cheshire UK; ^4^ Repertoire Labs Essex UK; ^5^ Institute of Applied Health Research, College of Medical and Dental Sciences University of Birmingham Birmingham UK; ^6^ Health Data Research UK London UK

**Keywords:** artificial intelligence, COVID‐19, rapid learning systems

## Abstract

**Introduction:**

We report a pathfinder study of AI/knowledge engineering methods to rapidly formalise COVID‐19 guidelines into an executable model of decision making and care pathways. The knowledge source for the study was material published by BMJ Best Practice in March 2020.

**Methods:**

The *PROforma* guideline modelling language and OpenClinical.net authoring and publishing platform were used to create a data model for care of COVID‐19 patients together with executable models of rules, decisions and plans that interpret patient data and give personalised care advice.

**Results:**

*PROforma* and OpenClinical.net proved to be an effective combination for rapidly creating the COVID‐19 model; the Pathfinder 1 demonstrator is available for assessment at https://www.openclinical.net/index.php?id=746.

**Conclusions:**

This is believed to be the first use of AI/knowledge engineering methods for disseminating best‐practice in COVID‐19 care. It demonstrates a novel and promising approach to the rapid translation of clinical guidelines into point of care services, and a foundation for rapid learning systems in many areas of healthcare.

## 1 COVID‐19 AND THE POLYPHONY PROJECT

The COVID‐19 emergency is a massive challenge to human expertise and organisation, but it is also widely recognised as an opportunity to demonstrate, test and improve medical technologies, including artificial intelligence (AI) techniques for delivering rapid learning systems. Over recent years, we have been developing a flexible methodology for creating executable models of specialist clinical expertise and a platform for sharing these models called OpenClinical (www.OpenClinical.net).^1^

OpenClinical is one of a number of efforts in recent years to use knowledge engineering and other techniques to formalise clinical guidelines as “executable knowledge.”^2^, ^3^ Although systematic reviews, clinical guidelines, etc, have been important tools of the evidence‐based medicine movement their impact in improving consistency and quality of care has been less than hoped for because they are disseminated purely as human readable content (eg, text, diagrams) in a traditional, often slow, publication and revision cycle. OpenClinical has made use of one particular approach to formalising clinical guidelines based on a specialised modelling language called *PROforma* ^4^, ^5^; we adopted this approach because it has been used and trialled successfully in many medical applications^6^ and we have wide experience using it.

**FIGURE 1:**
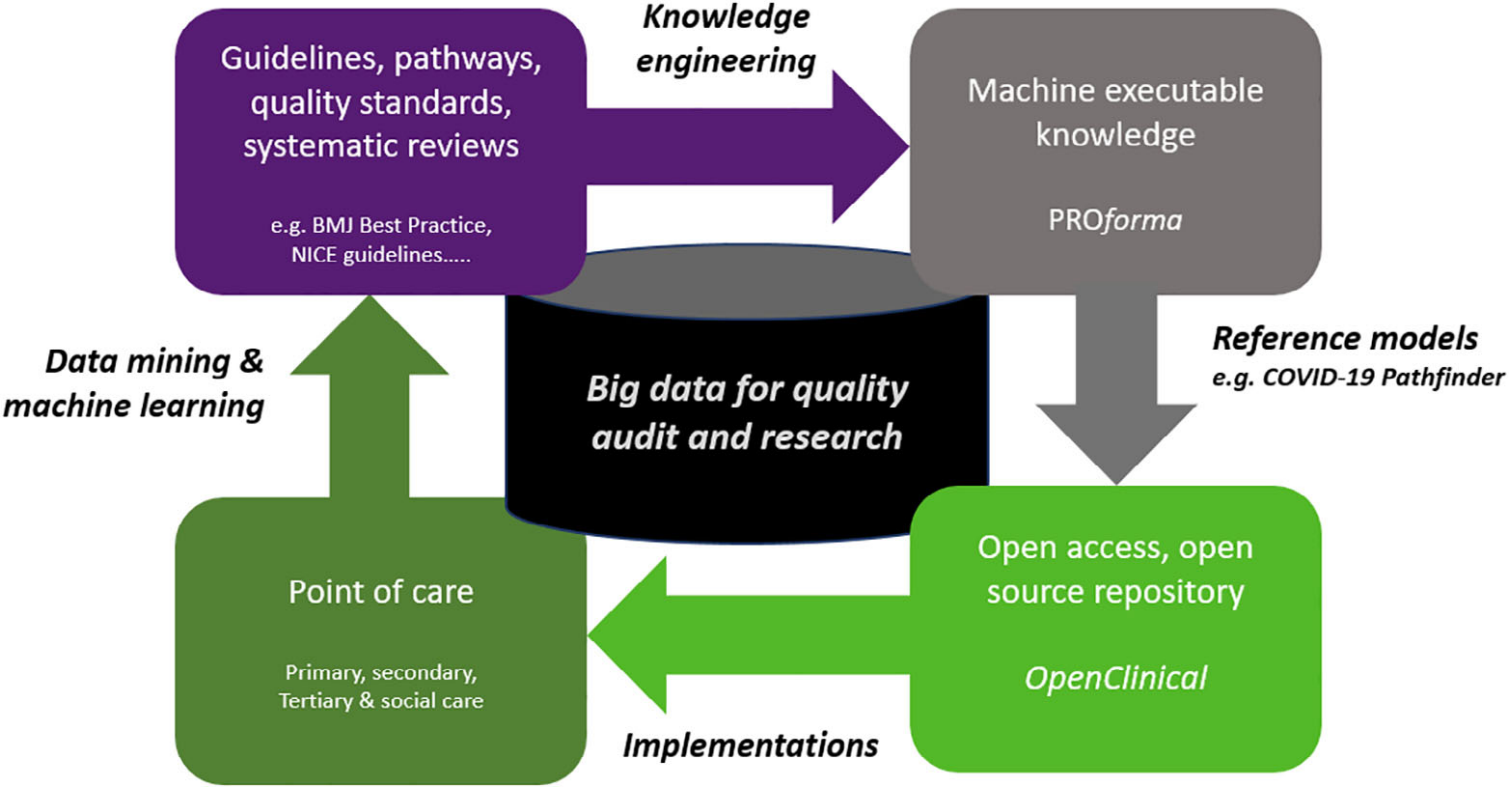
The OpenClinical knowledge‐to‐data cycle for rapid learning systems

We see OpenClinical as the basis of a rapid learning system as illustrated in Figure 1. This shows a knowledge life cycle for creating and maintaining executable models of care using the OpenClinical.net knowledge modelling and publishing platform. The top arrow of the cycle represents creation and testing of models using *PROforma* authoring software; the next step on the right is to publish them on the OpenClinical.net repository (which currently carries 50+ examples of executable models for many clinical settings and specialties, https://dev.openclinical.net/index.php?id=69).

This lifecycle overlaps with other Rapid Learning Systems (RLS) proposals, notably Friedman et al's^2^ MCBK proposals but is distinctive in using *PROforma*, a specialised AI language, for modelling and to support open source sharing of models. Although *PROforma* has been successful in these roles the goal of OpenClinical is primarily to provide a platform for crowdsourcing, validating and disseminating models not to deliver clinical services directly. A key aim of the OpenClinical project is to “close the loop” (left arrow) by incorporating software for acquiring clinical data from clinical implementations and trials of the models. This will permit use of data mining, machine learning and other techniques to update the evidence base and refine clinical guidelines and other care quality standards.

The Polyphony project was initiated on 18 March 2020 with the following mission.

To create, validate, publish and maintain knowledge of best medical practice regarding the detection, diagnosis and management of COVID‐19 infections, **in a computer executable form.** The purpose is to provide a resource for clinicians and researchers, healthcare provider organisations, technology developers and other users, to (1) develop point of care products and services which (2) embody best clinical practice in decision‐making, workflow, data analysis and other “intelligent” services across the COVID patient journey.

Among the questions we aimed to address in Polyphony are the following which relate directly to the knowledge lifecycle in Figure 1:

- Can we create executable models of best practice in the care of COVID‐19 patients? Can this demonstrate and maintain a standardised model of good practice (“reference model”)?
- How useful is the OpenClinical knowledge sharing framework for empowering clinicians to critique and improve models of decision‐making and care across the patient journey?
- Is it possible to adapt components of the model for use in different clinical settings or in local variants of care pathways?
- What is the potential for combining knowledge engineering methods with techniques from data science (eg, statistical analysis, data mining and machine learning)?

This article is a progress report on our first prototype, Pathfinder 1, and the design and engineering framework we have developed to support the Polyphony mission. The online demonstrator is not complete or “clinical strength” but this is the goal of future cycles of Figure 1.^*^ However sufficient progress has been made that it may be of interest to the RLS community.

## 2 METHODS

### 2.1 *PROforma*

The *PROforma* language is based on a general framework for modelling *tasks*, including reasoning, decision‐making and planning. Task models can be applied to knowledge about a particular medical domain such as the diagnosis and treatment of COVID‐19 patients, formalised in *PROforma* or available in external resources.

**FIGURE 2:**
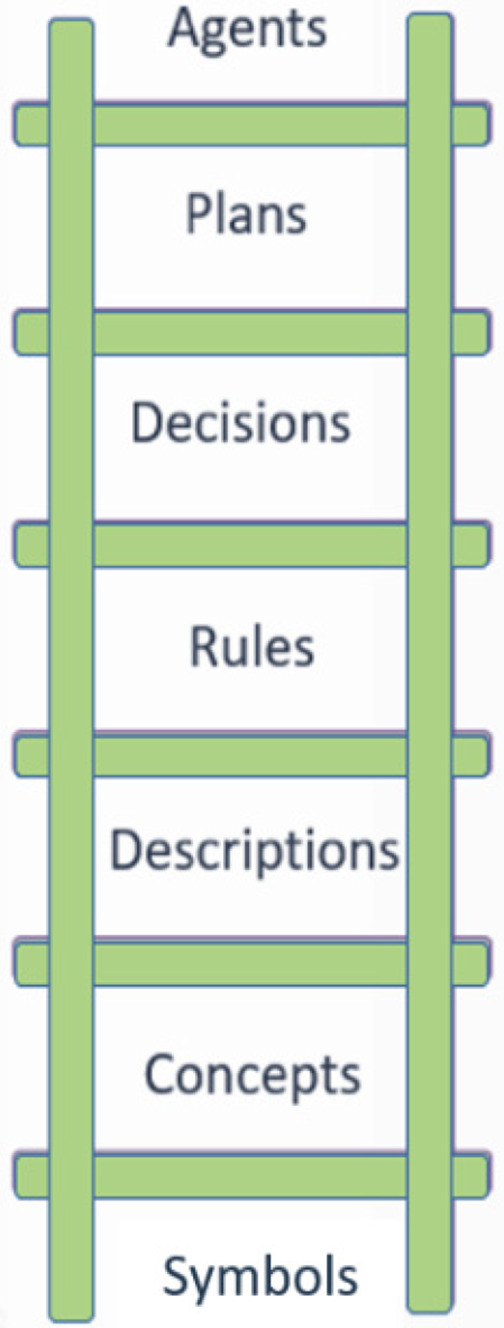
The *PROforma* knowledge ladder

The OpenClinical knowledge model is illustrated schematically in Figure 2. At the bottom of the ladder are *symbols* (eg, “fever,” “38.6”) which can be combined to represent data (eg, presenting symptoms include fever) and *concepts* (eg, diagnosis is a kind of decision; pathway is a kind of plan) and *descriptions* (eg, patient histories). *PROforma* can be used to model knowledge as rules for inference or action. Where inference is uncertain rules can participate in complex *decisions* as a basis for constructing *arguments* for and against competing decision options. Finally, *decisions, actions* and *enquiries* (actions that acquire information) can be composed into *plans* to achieve particular objectives. In principle plans can be encapsulated as *agents* that can carry out complex behaviour autonomously, though agent modelling is not within the scope of the Polyphony programme.

### 2.2 Knowledge modelling

*PROforma* can be used to model clinical guidelines of various types including medical logic and recommendations, decision trees, clinical algorithms, etc. Early development of the COVID‐19 knowledge base in Pathfinder 1 relied primarily on documentation published by *BMJ Best Practice* in March 2020.^†^ The OpenClinical modelling and testing platform was used by an experienced *PROforma* modeller advised by clinicians with specialist knowledge in public health and primary care. In Pathfinder 1 the concepts and components of the BMJ *Best practice* guidance were mapped directly to levels of the knowledge ladder. The resulting COVID‐19 model consists of five main sections: the data model; clinical contexts (data sets and scenarios); rules (inference and alerts); decisions (arguments and evidence); and care pathways. In the following paragraphs, we summarise how these are modelled in the various modules of Pathfinder 1. A module overview can also be seen in the graphic on the Pathfinder page on OpenClinical.

### 2.3 Data model

The OpenClinical platform includes a tool to create *data definitions* for relevant clinical and other parameters, their types (strings, integers, date‐times, etc) and other properties.^‡^ The full model for Pathfinder 1 consists of 46 data definitions based on the *BMJ Best Practice* documentation (March 2020). These are at the symbol level of the knowledge ladder in Figure 2.

### 2.4 Clinical contexts

A clinical context is typically a scenario on the patient journey in which one or more decisions may be taken and for which subsets of the data model are relevant. One scenario is “patient triage” where a handful of questions may be asked relevant to a decision whether to do nothing, advise self‐isolation or book an ambulance. Another context is “hospital work‐up” which covers a detailed patient history to inform a provisional diagnosis and initial selection of investigations. Based on the BMJ guidance 10 contexts were identified and modelled. Pathfinder 1 emphasises pre‐hospital scenarios with less emphasis on hospital and acute care, though by the time of publication BMJ Best Practice has added more guidance on the latter stages of the journey.

### 2.5 Rules: Inference and alerts

As shown in the knowledge ladder *PROforma* supports several knowledge types, of which the simplest is the *if…then…* rule. Rules respond to situations or events expressed in Boolean logic. For example, if a patient's temperature is >38.6 Pathfinder infers there is a fever; when hazardous situations are detected “red flags” are raised. Other uses of rules are to alert the user if a task has not been carried out or is overdue, or when a patient is eligible for inclusion in an open research trial.^7^

### 2.6 Decisions

A *PROforma* decision model consists of a set of *options* and a set of “arguments” (pros and cons) associated with each option. If an argument expression is evaluated against current patient data or other information and found to be valid, it is recorded as a *reason* for (or against) the relevant option with an explanation and, if required, supporting evidence that justifies the argument. All options in an active decision context accumulate patient‐specific pros‐ and cons as data are acquired, and the decision engine aggregates these to provide a continuously updated measure of confidence in each option. In the initial modelling stage arguments are usually modelled qualitatively but if statistical or other data are available they can be assigned quantitative weights which can be aggregated using various possible decision algorithms.

Eight main decisions have been modelled for the COVID‐19 patient journey, including triage, diagnosis, prognosis, prediction of complications and choice of management plan. All decisions are concurrently active in Pathfinder 1 but they can also be deployed at specific points or in particular scenarios in the care pathway.

### 2.7 Pathways

**FIGURE 3:**
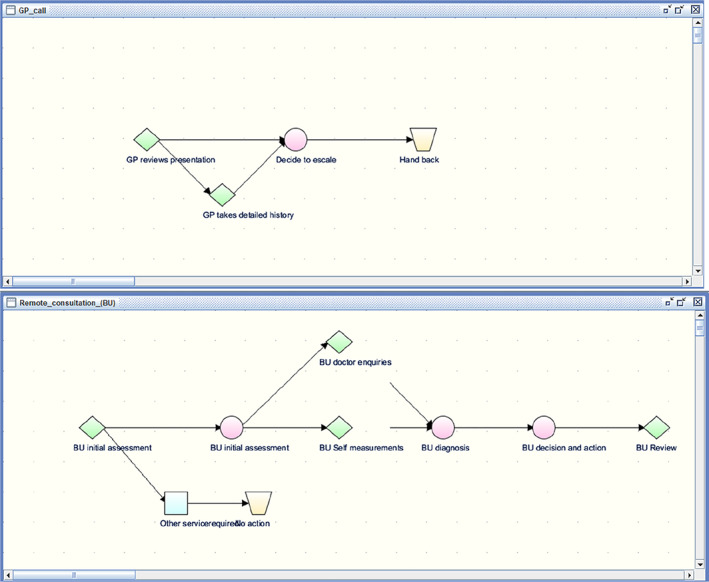
Two versions of remote consultation pathways for GP scenarios

A “pathway” is a network of decisions and tasks for acquiring data and carrying out plans in a sequential and/or conditional way as illustrated in Figure 3. In the first version there is an enquiry (green diamond) about a small number of key data relevant to an initial assessment (eg, presenting complaints, age of patient and whether patient seems ill). If required a more detailed history is taken but either way an escalation decision follows. A later version shown in the second panel is based on more detailed guidance to GPs published later by BMJ.

## 3 RESULTS

**FIGURE 4:**
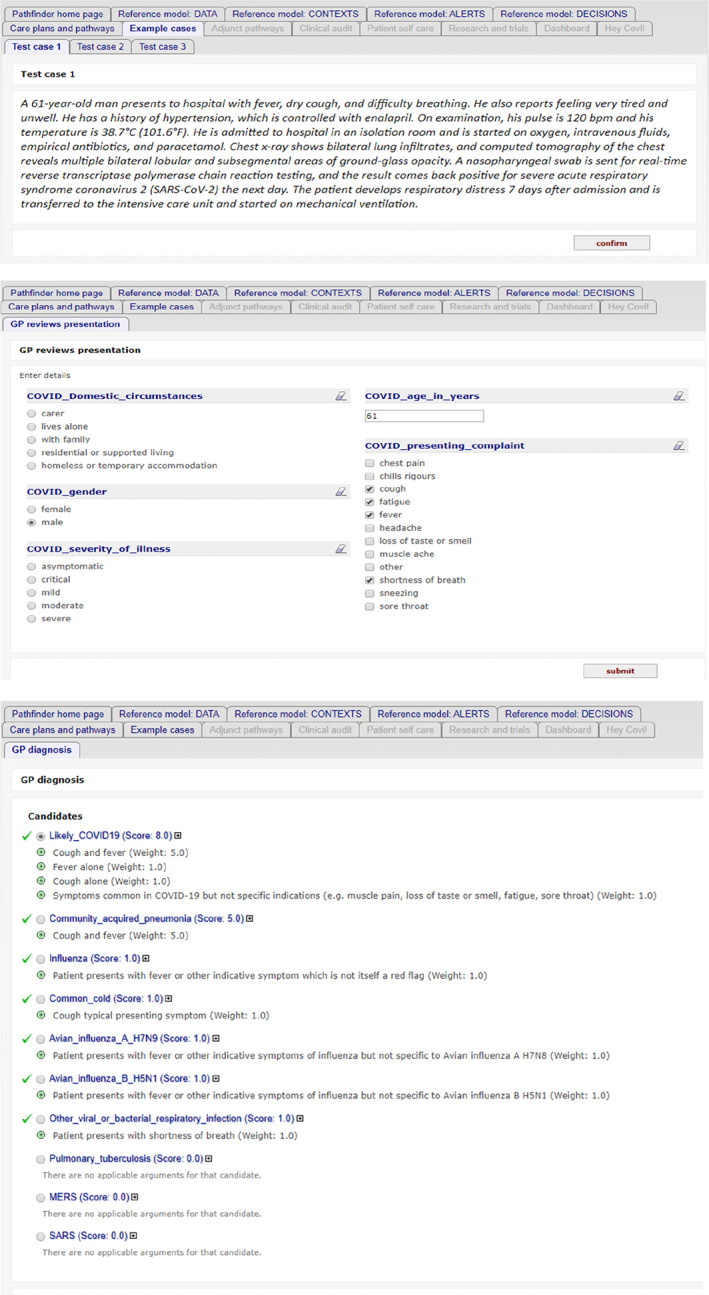
Screenshots of data and argumentation in the diagnosis decision, for example, case 1. A, Loading one of the patient test cases (ack. BMJ), B, a data entry form for a GP consultation, partly pre‐populated from the case, and C, arguments for different diagnoses based on presenting data

Pathfinder 1 can be accessed via the link https://www.openclinical.net/index.php?id=68, where there are instructions for running the demonstrator against example cases provided or against the user's own cases. Figure 4 shows three screenshots from a typical run against example case 1.

When running the online demonstrator the following points should be borne in mind: (a) The model is developed as a “standalone” resource for testing and validation and is not intended to be deployed directly into clinical use. The model is incomplete with respect to current COVID‐19 guidance which has evolved significantly since completion of the first modelling cycle; we expect to make significant revisions to the data model and knowledge content in light of experience and feedback before starting on the next modelling cycle (Pathfinder 2).

## 4 DISCUSSION

### 4.1 Assessment against project objectives

#### 4.1.1 Creation of an executable model of best practice for supporting care of COVID‐19 patients

*PROforma* is capable of modelling the main decisions and workflows across the COVID‐19 patient journey. Pathfinder was developed in about 3 weeks but is incomplete with respect to published medical knowledge and recommended practice and the data, decision and pathway models all require improvement and validation by qualified clinicians. Our provisional assessment is that the model could be completed and maintained on a rapid timescale. A complementary pathway for hospital care of COVID‐19 patients has also been rapidly developed in *PROforma* by Deontics Ltd.^§^ The focus of Pathfinder 2 is on extending the pathways for additional clinical contexts (eg, comorbidity management).

#### 4.1.2 Appraisal of the OpenClinical platform as a practical knowledge sharing infrastructure

Pathfinder 1 was deployed on OpenClinical.net as a globally accessible, open access demonstrator in early April 2020. A modified version was made in response to comments and suggestions of the clinical authors and made privately available for testing by them shortly after. The incorporation of example test cases provided by clinical collaborators and independent reviewers helps to quickly familiarise users with the demonstration and to critique the decision models and pathways against realistic patient data.

#### 4.1.3 Reusability of the data and knowledge models at different points in the care journey

The “escalation decision” in the reference model was initially used in a self‐triage pathway and reused in the residential care triage pathway of Pathfinder 1, but was replaced with three different decisions in the GP consultation pathway in light of clinical comments and new published guidance. In the latter pathway the diagnosis decision from the Pathfinder 1 model was reused without change, but significant changes were made to the initial assessment scenario and additional decisions about diagnosis and appropriate actions were added to the later pathway model.

#### 4.1.4 Scope for integrating data analysis and machine learning functions

At this early stage of the project, we have not been able to progress this question but are seeking to collaborate with data science specialists in the next cycle of the project. Initial discussions suggest, however, that once case records for a population of patients are available it will be practical to exploit well established statistical and machine learning methods to calibrate argument weights for patient populations, for example, and there is also scope for symbolic machine learning methods to suggest extensions to the logical knowledge model (eg, rule induction methods).

### 4.2 Deployments

As explained above the main goal of the Polyphony project is to develop a “reference” data and knowledge model, not to deploy the model directly but to be a resource for others to use in developing clinical services. This is in part due to the complexities of integrating decision support and other services with existing IT infrastructure. As mentioned above Deontics Ltd. have developed a decision support application for hospital patient assessment and deployed it in the emergency department of the Liverpool and Broadgreen University Hospital. Integration with the hospital EMR and infrastructure has been achieved but was significantly more complex than the *PROforma* modelling step.

Readers interested in deployment of services will ask how *PROforma* engages with relevant technical standards, such as medical coding and terminology standards (eg, ICD10; LOINC), ontologies (eg, SNOMED CT, UML), logical expressions (eg, GELLO) and rules (eg, Arden Syntax; CQL). *PROforma* overlaps conceptually but does not comply with these standards. The emphasis in *PROforma* is on capturing knowledge of these and other kinds in a single language for which it offers a unified syntax and execution semantics.^4^ This is highly preferable to modelling a complex body of knowledge with ad hoc combinations of notations.^8^

The development of HL7 FHIR resources has a similar motivation and we are interested in exploring whether *PROforma* can implement certain classes of FHIR resources, such as the FHIR plan definition (https://www.hl7.org/fhir/plandefinition.html) and FHIR executable knowledge artifact (https://www.hl7.org/fhir/clinicalreasoning-knowledge-artifact-representation.html). We think that this may make integration and deployment easier without losing the simplicity and intuitiveness of the *PROforma* and Polyphony framework.

If the Polyphony model is to have a sustainable future it will need to be repeatedly updated as knowledge of COVID‐19 and current clinical practice change. Maintenance can take place at two levels, the implementation (eg, software) level and the practice (knowledge) level.

We anticipate that the maintenance of the implementation can be managed through standard software engineering and version control methods. This allows messages and documentation outlining the rationale for the change to be associated with each iteration of the implementation. Present version control tools also keep a history of all changes, including highlighting differences between versions for easy review/audit. Classical version control would, for example, be appropriate at the level of HL7 resources and their mappings to the knowledge model.

Maintenance at the knowledge level is likely to be very different because the principle stakeholders here are professional clinicians, researchers and other subject matter experts. Polyphony was established under the auspices of the OpenClinical knowledge sharing project which seeks to establish a framework for knowledge dissemination analogous to open access publishing. Key here are the need for models to be intuitive and open to review and criticism by professional who have little expertise or interest in technicalities. New versions of knowledge models need to specify their provenance, the evidence on which changes are based and in clinical use any unexpected behaviour must be explainable in appropriate medical terms.

### 4.3 Wider questions

If COVID‐19 remains clinically challenging in the medium‐term it would be desirable to recruit experienced healthcare professionals to contribute to modelling the knowledge underlying decisions and pathways. Through OpenClinical we hope to support a sustainable community of practice to promote discussion and debate and to own and maintain the reference knowledge base. A possible organisational structure could be analogous to the “chromosome committees” of the human genome project in that specialist professional groups of GPs, emergency medicine clinicians, etc. would take responsibility for data and knowledge modelling in specific contexts along the patient journey, but adopting common data and knowledge representation standards.

In the longer term, with the hoped‐for arrival of an effective vaccine or treatments, the COVID‐19 emergency may pass or become tolerated as a seasonal burden like flu. These futures are controversial, and we take no position on them, but it is widely accepted that the COVID‐19 pandemic is only the latest in a series of infections with major consequences for human populations and there will be more to come. It will be important to have “rapid response” as well as “rapid learning” mechanisms in place. Polyphony may help to inform the design of policies and mechanisms by which expert and experienced healthcare professionals can form rapid response teams to address emerging threats.

A longer‐term objective is based on the proposition that the Polyphony approach is not limited to the COVID‐19 emergency nor even only to infections. The methods outlined here are applicable to rapid deployment of executable clinical guidelines and quality standards generally. We believe they can be used to create open access and open source models of practice for many conditions whether acute or chronic, commonplace or rare, “from home to hospital to home”.

## CONFLICT OF INTEREST

John Fox is founder and non‐executive director of Deontics Ltd. He also works pro bono as director of OpenClinical CIC, a non‐profit community interest company. Joht S. Chandan, Omar Khan, Jennifer Cooper, Neil Cockburn, Krishnarajah Nirantharakumar and Carla Pal state that they have no conflicts of interest. Both Hywel Curtis and Andrew Wright, who worked in advisory capacities as consultants on the project, also assert they have no conflicts of interest.

## Endnotes

^*^ As medical knowledge is constantly advancing there will never be a complete model. This is particularly clear in the case of COVID‐19 where the pace of advance and changes in practise are so extraordinarily rapid.

^†^ Thanks to Kieran Walsh and Chris Wroe at *BMJ Best practice* for permission to use the BMJBP article on COVID‐19 as the source material for this project (https://bestpractice.bmj.com/topics/en-gb/3000168). The content has been continuously updated by BMJ since then.

^‡^ There are a number of commercial *PROforma* authoring tools available including Arezzo (Elsevier) and Alium Composer (Deontics).

^§^ Declaration of interest: Deontics Ltd. is a spinout from the University of Oxford, University College London and Cancer Research UK. J. F. is a founder and holds equity in the company.
